# Self-medication with proton pump inhibitors among physicians in Bosnia and Herzegovina: a cross-sectional study

**DOI:** 10.3389/fmed.2026.1663331

**Published:** 2026-02-10

**Authors:** Predrag Jovanovic, Goran Bokan, Mirela Basic Denjagic, Nermin Salkic, Admir Kurtcehajic, Aleksandar Gavric, Kenana Ljuca, Mario Tadic, Rusmir Mesihovic, Goran Hauser, Dina Zerem

**Affiliations:** 1 Department of Gastroenterology and Hepatology, University Clinical Center Tuzla, Tuzla, Bosnia and Herzegovina; 2 Department of Internal Medicine, Faculty of Medicine, University of Tuzla, Tuzla, Bosnia and Herzegovina; 3 Internal Medicine Clinic, Department of Gastroenterology and Hepatology, University Clinical Centre of the Republic of Srpska, Banja Luka, Bosnia and Herzegovina; 4 Faculty of Medicine, University of Banja Luka, Banja Luka, Bosnia and Herzegovina; 5 Department of Gastroenterology and Hepatology, University Medical Center Ljubljana, Ljubljana, Slovenia; 6 Department of Gastroenterology and Hepatology, Medical Center Plava Poliklinika, Tuzla, Bosnia and Herzegovina; 7 Department of Gynecology and Obstetrics, University Clinical Center Ljubljana, Ljubljana, Slovenia; 8 Faculty of Pharmacy and Biochemistry, University of Zagreb, Zagreb, Croatia; 9 Department of Gastroenterology, Hepatology and Clinical Nutrition, Clinical Hospital Dubrava, Zagreb, Croatia; 10 Merus Medicinski Center, Ljubljana, Slovenia; 11 Department of Gastroenterology, CHC Rijeka, Rijeka, Croatia; 12 Faculty of Medicine, University of Rijeka, Rijeka, Croatia; 13 Cantonal Hospital “Dr. Safet Mujic,” Mostar, Bosnia and Herzegovina

**Keywords:** cross-sectional study, physicians, prescribing behavior, proton pump inhibitors, real-world evidence, self-medication

## Abstract

**Background:**

Proton pump inhibitors (PPIs) are widely used for the treatment of acid-related disorders, but inappropriate or prolonged use carries potential health risks. Physicians, due to their access to medication and clinical knowledge, may be prone to self-medicating with PPIs without appropriate oversight.

**Objective:**

To assess the prevalence and patterns of personal PPI use and self-medication among practicing physicians in Bosnia and Herzegovina, and to identify demographic and professional predictors of such behavior.

**Methods:**

A cross-sectional, questionnaire-based survey was conducted among 448 physicians who responded to the study invitation, out of approximately 600 invited, from various healthcare levels in Bosnia and Herzegovina between January and May 2025. The survey collected data on PPI use history, consultation behavior, awareness of adverse effects, and adherence to treatment guidelines. Multivariable logistic regression was used to identify independent predictors of self-medication.

**Results:**

A total of 65.4% of respondents reported past PPI use, during their medical practice, and 31.7% were current users. Over half (52.2%) admitted using PPIs without consulting another physician, and only 17.4% referred to clinical guidelines prior to use. Occasional use was the most common pattern (59.0%), while adverse effects were rarely reported (1.8%). No demographic or professional variable was significantly associated with self-medication with PPIs (defined as PPI use without consulting another physician) in the multivariable analysis.

**Conclusion:**

Self-medication with PPIs is highly prevalent among physicians and frequently occurs without clinical consultation or adherence to guidelines. This behavior appears to be widespread across age groups, sexes, and care levels, highlighting the need for institutional interventions that promote rational prescribing and raise awareness about responsible self-care within the medical profession.

## Introduction

Proton pump inhibitors (PPIs) are commonly prescribed for acid-related gastrointestinal conditions, including gastroesophageal reflux disease, peptic ulcer disease, and dyspepsia. Although considered safe for short-term use, concerns have been raised regarding their overprescription and inappropriate long-term use (1). Prolonged therapy has been linked to adverse outcomes such as nutrient deficiencies, gastrointestinal infections, bone fractures, renal impairment, and potential neurological effects (2). In response, clinical guidelines increasingly emphasize indication-based prescribing and periodic reassessment.

Paradoxically, physicians themselves may contribute to this trend. Their dual role as prescribers and potential consumers facilitates easy access to medications and may foster informal self-treatment. Evidence suggests that physicians often bypass formal medical consultation, relying instead on perceived clinical knowledge and experience (3).

While self-medication among healthcare professionals has been explored in the context of antibiotics, anxiolytics, and analgesics, little is known about this behavior regarding PPIs. Given their over-the-counter availability and low perceived risk, PPIs may be particularly prone to unsupervised use within the medical community.

This study aimed to investigate the prevalence and patterns of personal PPI use among physicians in Bosnia and Herzegovina and to identify demographic and professional predictors of self-medication. Understanding these behaviors may inform institutional strategies to reinforce rational prescribing and promote responsible self-care among healthcare providers.

## Materials and methods

A cross-sectional, questionnaire-based study was conducted among licensed physicians in Bosnia and Herzegovina between January and May 2025. Eligible participants included physicians from all levels of healthcare (primary, secondary, tertiary, and private practice) and across various specialties, provided they were actively involved in clinical care at the time of the study.

Data were collected using a structured, self-administered online questionnaire distributed via Google Forms. The survey link was disseminated through institutional mailing lists and professional academic meetings. Approximately 600 physicians were invited to participate, representing about 7.5% of the national physician workforce in Bosnia and Herzegovina (approximately 8,000 licensed physicians, based on publicly available national healthcare workforce data), and 448 completed the questionnaire (response rate ≈ 75%). A convenience sampling approach was used across all major healthcare levels and specialties. Participation was voluntary and anonymous, with no incentives provided.

The questionnaire consisted of 30 closed-ended items grouped into four thematic domains:

(1)    Demographic and professional characteristics
(2)    Personal use and usage patterns of PPIs
(3)    Consultation behavior, access, and oversight
(4)    Awareness of adverse effects and familiarity with treatment guidelines

The questionnaire was developed specifically for this study, drawing on items used in previously published surveys on physicians’ self-medication and PPI use. The instrument was piloted with 10 physicians and reviewed by three senior gastroenterologists to ensure content and face validity. No further psychometric validation (such as internal consistency or reliability testing) was performed, as the questionnaire consisted of categorical descriptive items rather than latent-scale constructs. Additionally, traditional reliability indices (such as Cronbach’s α) were not applicable, since the questionnaire did not include multi-item scales designed to measure underlying psychometric constructs, but instead comprised standalone categorical variables.

The original questionnaire (in Bosnian/Serbian/Croatian) and its verbatim English translation are provided as Supplementary Material 1.

Descriptive statistics were used to summarize respondent characteristics and patterns of PPI use. Associations between categorical variables were analyzed using Chi-square or Fisher’s exact test, as appropriate. The Chi-square test was used for comparisons with adequate expected cell frequencies, while Fisher’s exact test was applied in cases of small expected cell counts (<5 in any cell). A multivariable logistic regression model was applied to identify independent predictors of PPI self-medication, defined as use without consultation with another physician. Predictor variables included age under 35 years, sex, and tertiary-care employment. Age was included as a categorical variable (<35 vs. ≥35 years), and all predictor variables were dummy coded (binary), with reference categories defined as age ≥ 35, male sex, and non–tertiary-care employment. For the multivariable logistic regression, we a priori selected age, sex, and healthcare level (tertiary vs. non-tertiary) as predictors to reflect demographic and workplace determinants of self-medication; clinical variables such as GERD diagnosis and body weight, which primarily indicate the need for PPI therapy rather than the likelihood of unsupervised use, were not included as candidate predictors. Predictor variables were dichotomized to ensure adequate cell counts for statistical stability and to test specific hypotheses regarding early-career status (Age < 35 vs. ≥35) and academic practice setting (Tertiary vs. Non-Tertiary).

Statistical analysis was performed using JASP software (version 0.19.1; Amsterdam, the Netherlands). A two-sided *p*-value of <0.05 was considered statistically significant.

## Results

A total of 448 physicians completed the survey. The sample was predominantly female (63.2%), with the largest age group being 36–50 years (51.8%), followed by those under 35 years (29.9%). More than half were employed in tertiary care institutions (52.5%), while the remainder worked in primary care (19.9%), secondary hospitals (17.4%), and private practices (10.3%).

Overall, 65.4% of respondents reported previous PPI use at any point in their professional or personal history of medication use (“ever-use”), and 31.7% were current users. Pantoprazole was the most frequently used agent. The most common usage pattern was occasional, symptom-triggered intake (59.0%), followed by preventive use (6.0%), defined as taking PPIs in anticipation of triggers such as dietary indiscretion.

**FIGURE 1:**
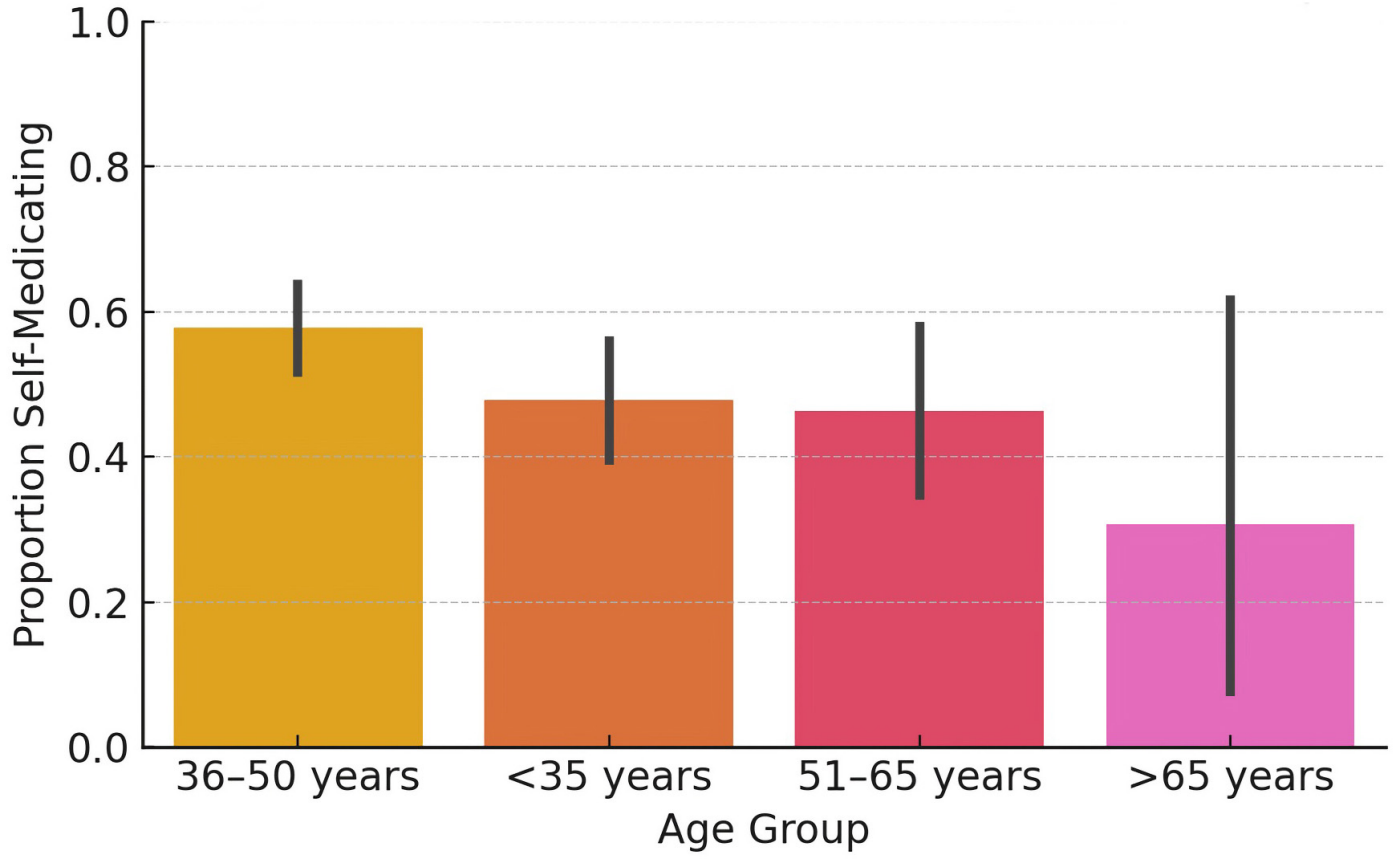
Proton pump inhibitor (PPI) self-medication by age group. Bar chart showing the proportion of physicians who reported using proton pump inhibitors (PPIs) without prior consultation, stratified by age group. Younger physicians (<35 years) exhibited slightly higher rates of self-medication compared to older groups.

**FIGURE 2:**
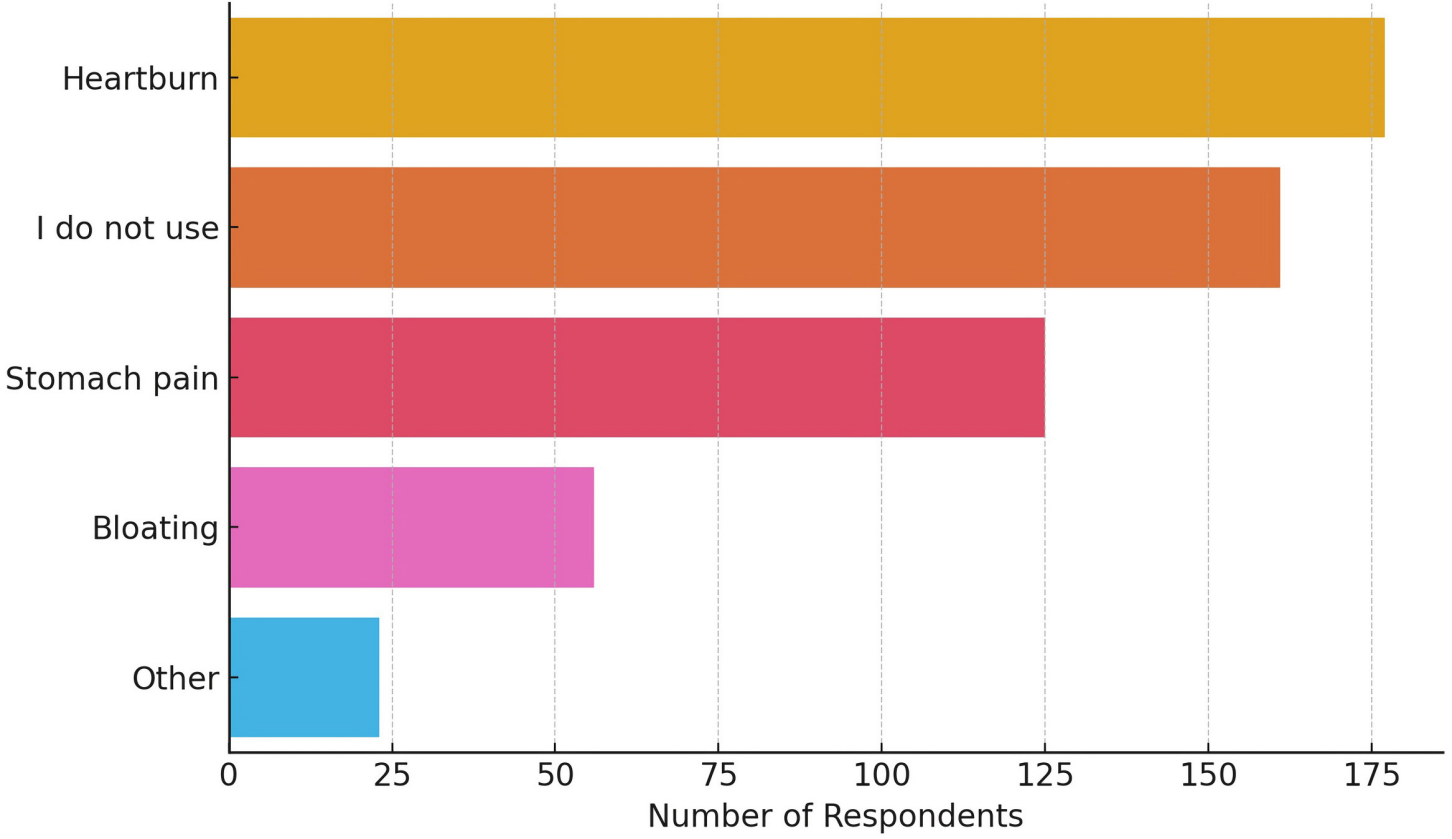
Reasons for occasional PPI use. Heartburn and stomach pain were the most frequently cited indications, followed by bloating and other less common complaints. The category “I do not use” reflects respondents who reported no symptomatic trigger for taking PPIs and is included as a distinct response option.

Self-medication–defined as use without consulting another physician–was reported by 52.2% of participants (Figure 1). Among those, only 17.4% consulted clinical guidelines prior to initiating therapy. Reasons for occasional PPI use are presented in Figure 2.

Adverse effects were infrequent (1.8%), with diarrhea and headache being the most commonly reported symptoms. Most respondents did not report any adverse effects, which may reflect both the generally favorable safety profile of PPIs and a tendency among physicians to overlook or underreport mild, self-limited symptoms.

**FIGURE 3:**
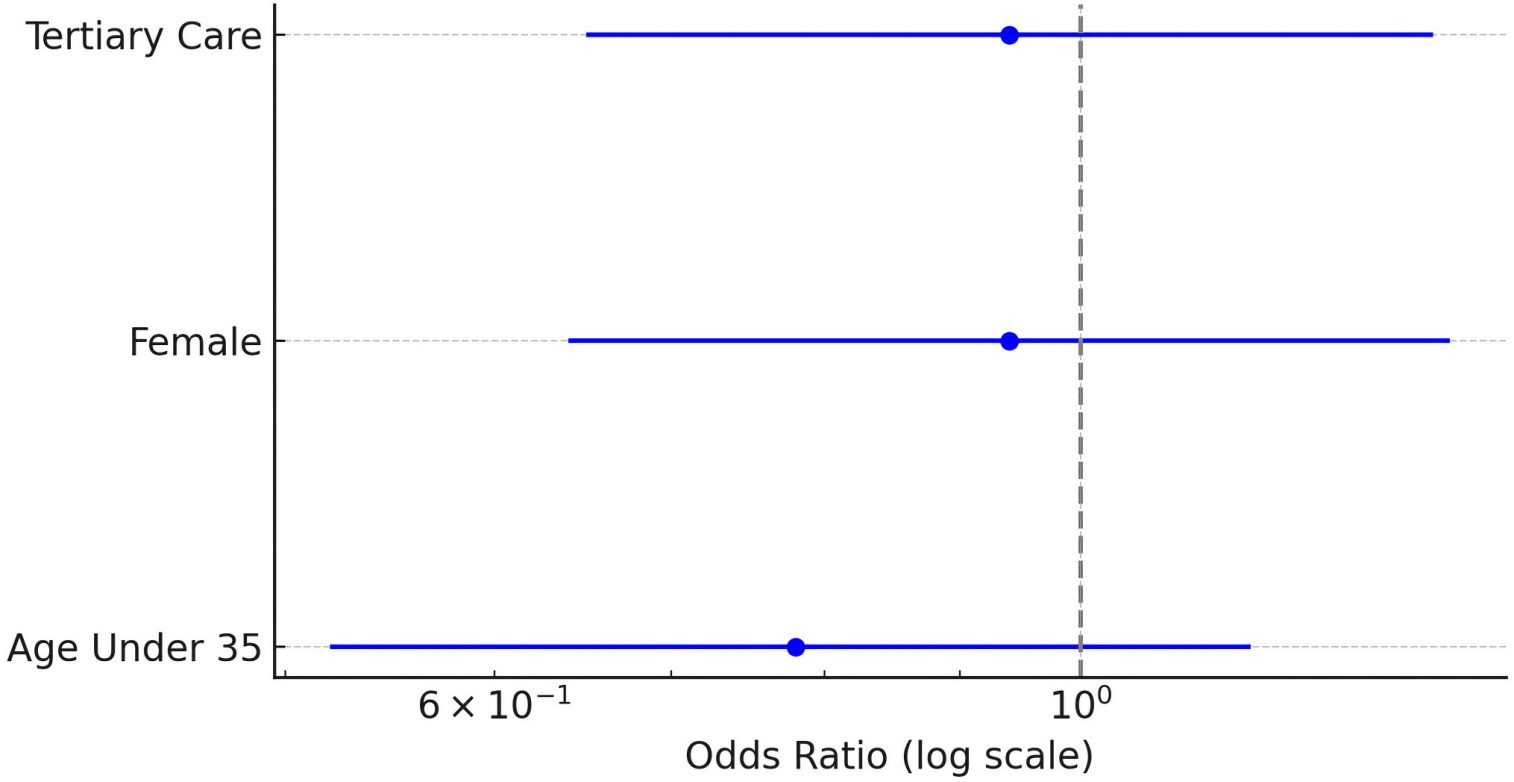
Self-medication proportions by age group and level of healthcare. Age categories correspond to <35, 36–50, 51–65, and >65 years, although the visual ordering reflects default plotting output. Cells with no respondents are uncolored, whereas white-shaded cells represent respondent groups in which zero self-medication was reported. This distinction supports accurate interpretation of subgroup variation, particularly in the >65 age group across care levels.

**FIGURE 4:**
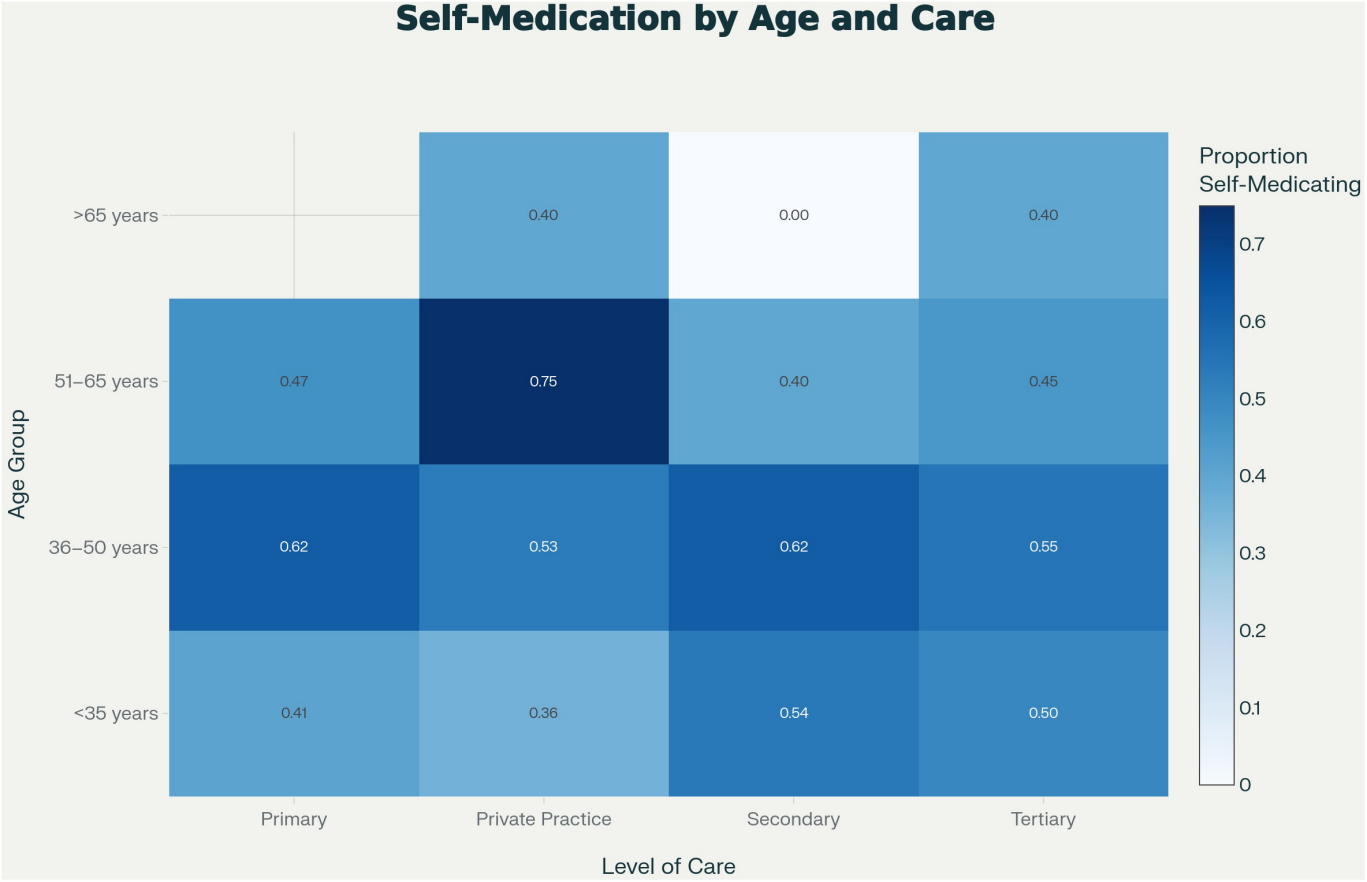
Adjusted odds ratios for self-medication with PPIs. Forest plot displaying multivariable logistic regression results with odds ratios and 95% confidence intervals for key demographic and professional predictors. Age < 35, female sex, and tertiary-care employment were coded as dummy variables with age = 35, male sex, and non-tertiary care serving as reference categories. OR = 1.0 indicates no association.

Subgroup analysis showed higher rates of self-medication among younger physicians and those working in tertiary care institutions (Figure 3). However, none of these associations were statistically significant in the multivariable logistic regression model. Specifically, being under the age of 35 was associated with an adjusted odds ratio (OR) of 0.78 (95% CI: 0.52–1.16; *p* = 0.22). Female sex (OR = 0.94; *p* = 0.75) and tertiary-care employment (OR = 0.94; *p* = 0.74) were also not significant predictors (Figure 4).

**FIGURE 5:**
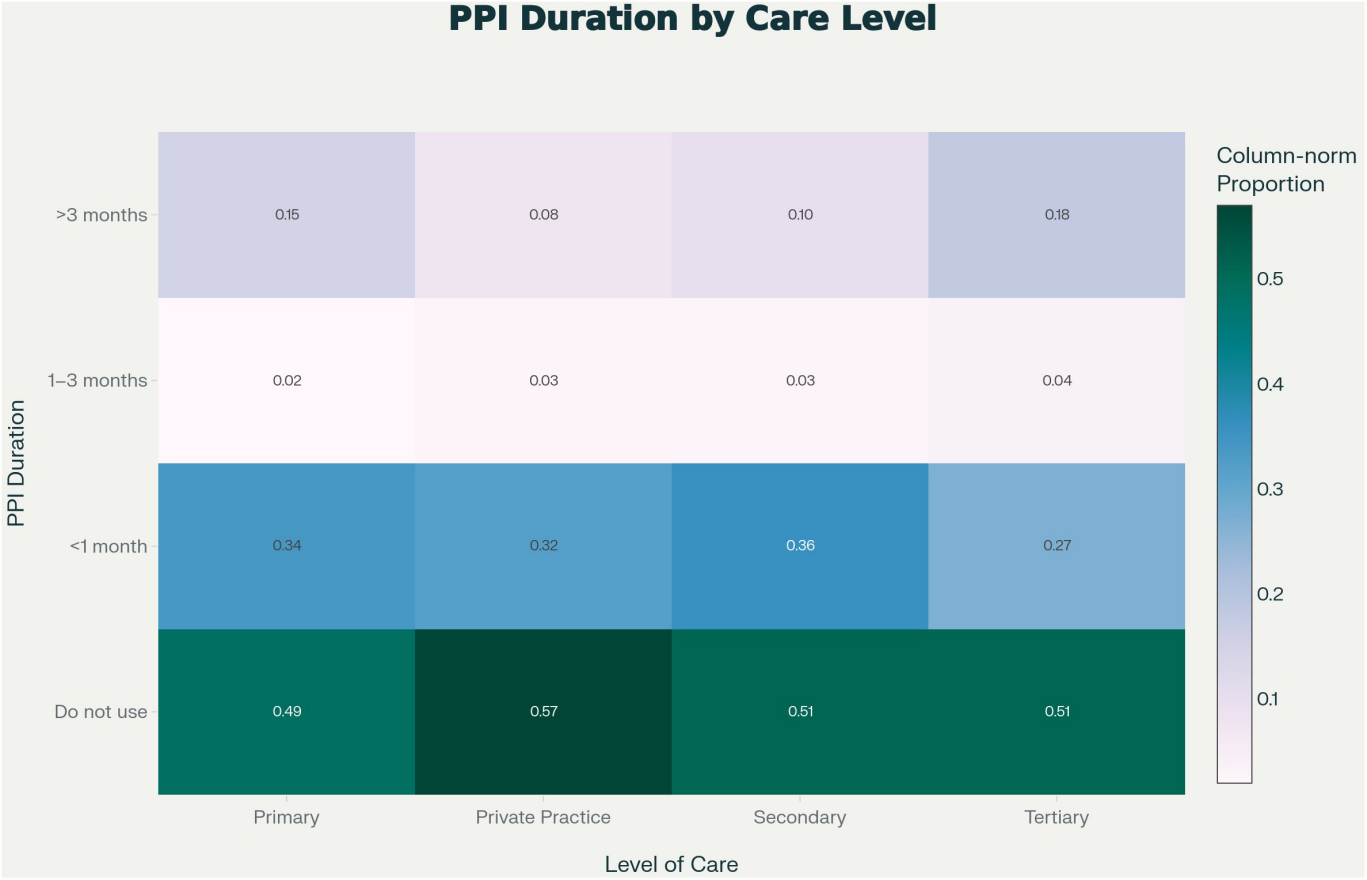
Duration of self-medication by level of care. Heatmap showing the duration of unsupervised PPI use across different care levels. Responses indicate variability in short-term versus long-term use, with some clustering of prolonged use in tertiary and private practice settings. This pattern suggests that physicians working in more specialized environments may be more inclined to extend therapy beyond standard short-term indications.

The duration of unsupervised PPI use across care levels is shown in Figure 5. Most physicians reported using PPIs for less than 1 month, consistent with episodic symptom management, whereas prolonged use (>3 months) was more frequent among those in tertiary and private practice settings. This may reflect easier access to medications and lower perceived risk of long-term use among physicians in these environments.

## Discussion

### Principal findings

This study demonstrates that self-medication with proton pump inhibitors (PPIs) is highly prevalent among physicians in Bosnia and Herzegovina. More than half of the surveyed participants reported using PPIs without medical consultation, and only a minority consulted clinical guidelines. These findings are consistent with international data suggesting that physicians are prone to informal pharmacotherapy, often bypassing formal evaluation due to perceived familiarity with medications and disease mechanisms (3, 4).

Although PPIs are considered safe, their inappropriate prolonged use - especially without clear indication - has been associated with significant risks, including gastrointestinal infections, renal impairment, and micronutrient deficiencies (2, 5). Despite these risks, adverse effects were rarely reported in our cohort, potentially due to underrecognition or the relatively short duration of use. This gap between knowledge and behavior may reflect cognitive biases and normalization of informal treatment practices within the medical profession.

Our regression analysis did not identify any statistically significant predictors of self-medication. While younger physicians and those in tertiary care appeared more likely to self-medicate, these associations were not confirmed in adjusted models. This suggests that PPI self-use is a widespread and normalized behavior across demographic and professional strata, consistent with prior findings from other health systems (6, 7).

However, the tendency toward prolonged self-medication observed among physicians in tertiary and private practice settings may indicate a perceived professional immunity to potential adverse effects, reflecting similar behavioral patterns reported in other healthcare systems.

The observed patterns of occasional, short-term PPI use were dominant across all levels of care, consistent with transient symptom management rather than sustained therapy. A smaller proportion of physicians, particularly in tertiary and private settings, reported prolonged use beyond 1 month. While not representative of the majority, this subgroup may reflect a tendency toward normalization of unsupervised longer-term therapy in certain professional environments. Importantly, a portion of reported PPI use appears to have been driven by transient, nonspecific upper gastrointestinal discomfort rather than by a guideline-supported indication such as confirmed GERD or peptic ulcer disease. This distinction is clinically relevant, as empiric use for vague symptoms without diagnostic evaluation may contribute to unnecessary exposure to PPI therapy.

### Comparison with other studies

In a recent study among healthcare professionals in Spain, Cotobal-Calvo et al. reported high rates of unsupervised medication use, underscoring the prevalence of this behavior in European contexts (6). Similarly, Al-Omrani et al. documented widespread self-medication among Saudi healthcare workers, emphasizing the global nature of the issue (7). Together with our findings, these studies highlight the need for tailored interventions that acknowledge both cultural and systemic factors influencing physician behavior.

A recent study among resident doctors in India highlighted similar gaps in knowledge and inconsistent adherence to PPI prescribing principles, even among early-career clinicians (8). Kurlander et al. found that many physicians acknowledge risks associated with PPI overuse but rarely act on deprescribing, pointing to a disconnect between awareness and behavior (9). Similar concerns about underrecognized adverse drug events have been raised in antibiotic stewardship literature, suggesting that this may reflect a broader issue in prescribing culture (10).

### Strengths and limitations

This study has limitations. It relied on self-reported data, which may be subject to recall bias or social desirability bias. The online survey format may have excluded physicians with limited digital access or motivation. Additionally, the cross-sectional design precludes any inference about causality or directionality between demographic factors and self-medication behaviors. A further limitation is that the questionnaire did not undergo formal psychometric reliability testing (e.g., internal consistency metrics), as its items were categorical and independent rather than scale-based; however, content and face validity were ensured through expert review and pilot testing. Furthermore, the convenience sampling approach may limit the generalizability of the findings to all physicians, although the inclusion of respondents from diverse healthcare levels partially mitigates this limitation.

### Implications for practice and research

Several implications emerge from these findings. First, the dissemination of clinical guidelines alone may be insufficient to influence personal prescribing habits. Educational interventions should address behavioral, institutional, and cultural dimensions, including the cognitive dissonance between physicians’ professional standards and their self-care behavior. Second, self-medication among healthcare professionals should be incorporated into institutional stewardship strategies. This could include confidential audits, peer accountability systems, and integration into continuing medical education (CME) curricula (11–13).

Importantly, physicians serve as both stewards and users of pharmacotherapy. Addressing informal medication use in this group is essential not only for individual safety but also for reinforcing a broader culture of rational prescribing.

## Conclusion

Self-medication with proton pump inhibitors is common among physicians and often occurs without professional consultation or adherence to clinical guidelines. This behavior appears normalized across age groups and healthcare levels, reflecting a disconnect between medical knowledge and personal practice. Institutional efforts to promote rational prescribing should include the self-care behavior of healthcare providers. Addressing this silent trend is critical for advancing pharmacologic stewardship and reinforcing professional accountability within the medical community.

## Data Availability

The raw data supporting the conclusions of this article will be made available by the authors, without undue reservation.
