# Supplementary material for: Self-medication with proton pump inhibitors among physicians in Bosnia and Herzegovina: a cross-sectional study
Source: Front Med (Lausanne). 2026 Feb 10;13:1663331. doi: 10.3389/fmed.2026.1663331 (PMC12929428; doi:10.3389/fmed.2026.1663331)
Supplement: Supplementary file 1 [file Supplementary_file_1.docx]

# Supplementary Material 1. Questionnaire (English translation)

Note: This is a direct English translation of the original questionnaire administered in Bosnian/Serbian/Croatian language.

1. Gender*

- - Male
- - Female

1. Age group*

- - <35 years
- - 36–50 years
- - 51–65 years
- - >65 years

1. Body weight*

- - <60 kg
- - 60–75 kg
- - 76–85 kg
- - >86 kg

1. Level of healthcare employment*

- - Primary care
- - Secondary care
- - Tertiary care
- - Private practice

1. Do you currently have or have you ever had a diagnosis of gastroesophageal reflux disease (GERD)?*

- - Yes, currently
- - Yes, in the past
- - No

1. Do you currently have or have you ever had a diagnosis of peptic ulcer disease (stomach or duodenal ulcer)?*

- - Yes, currently
- - Yes, in the past
- - No

1. Do you use nonsteroidal anti-inflammatory drugs (NSAIDs)?*

- - Yes, daily as part of regular therapy
- - Yes, occasionally
- - No

1. Do you use anticoagulant medications?*

- - Yes, daily as part of regular therapy
- - Yes, occasionally
- - No

1. Do you have chronic dyspeptic symptoms (stomach pain, bloating, heartburn)?*

- - Yes, most of the time
- - Yes, occasionally
- - No

1. Have you previously used proton pump inhibitors (PPIs)?*

- - Yes, as regular therapy
- - Yes, occasionally
- - No

1. Which PPI do you currently use as part of your regular therapy?*

- - Omeprazole
- - Pantoprazole
- - Lansoprazole
- - Esomeprazole
- - Other
- - I do not currently use a PPI

1. How long have you been using PPIs?*

- - <1 month
- - 1–3 months
- - 3–6 months
- - >6 months
- - I do not use them

1. What is your usual PPI dose?*

- - 10 mg
- - 20 mg
- - 30 mg
- - 40 mg
- - Other:
- - I do not use it

1. How many times per day do you take PPIs?*

- - Once daily
- - Twice daily
- - More than twice daily
- - I do not take it

1. Do you take PPIs before or after meals?*

- - Before meals
- - After meals
- - I do not take it

1. Do you use other acid-suppressive drugs (e.g., antacids, H₂ blockers)?*

- - Yes, regularly
- - Yes, occasionally
- - No

1. Do you have any of the following comorbidities?*

- - Diabetes
- - Hypertension
- - Chronic obstructive pulmonary disease (COPD)
- - Heart failure
- - Other
- - No

1. Do you have kidney disease/impairment?*

- - Yes
- - No

1. Do you have osteoporosis?*

- - Yes
- - No

1. Do you have or have you had gastrointestinal infections (e.g., Clostridium difficile)?*

- - Yes
- - No

1. Do you use PPIs occasionally (as needed)?*

- - Yes
- - No

1. How often do you use PPIs on an occasional basis?*

- - Less than once a month
- - Once a month
- - Once a week
- - More than once a week
- - I do not take PPIs

1. What are the main reasons for your occasional PPI use?* (check all that apply)

- - Heartburn
- - Stomach pain
- - Bloating
- - Other
- - I do not use them

1. Do you use PPIs without prior consultation with a physician/gastroenterologist?*

- - Yes
- - No

1. If you self-medicate with PPIs, for how long have you used them without medical advice?*

- - Less than one month
- - 1–3 months
- - More than 3 months
- - I do not self-medicate

1. Do you use PPIs as preventive therapy (e.g., before consuming fatty food or alcohol)?*

- - Yes
- - No

1. Have you experienced inadequate therapeutic effect of one PPI and the need to switch to another?*

- - Yes
- - No

1. Have you experienced any adverse effects related to PPI therapy?*

- - Yes
- - No

1. Have you ever reduced the dose or discontinued PPI therapy due to adverse effects?*

- - Yes
- - No

1. What was the most common adverse effect you experienced during PPI therapy?*

- - Diarrhea
- - Constipation
- - Nausea
- - Abdominal pain
- - Other
- - I did not experience any
